# Recurrent Herpetic Stromal Keratitis in Mice: A Model for Studying Human HSK

**DOI:** 10.1155/2012/728480

**Published:** 2012-04-23

**Authors:** Patrick M. Stuart, Tammie L. Keadle

**Affiliations:** ^1^Department of Ophthalmology, Saint Louis University School of Medicine, 1402 S. Grand Blvd., Schwitalla Hall, Room M99, St. Louis, MO 63104-1004, USA; ^2^Department of Biology, Washington University School of Medicine, St. Louis, MO 63104, USA

## Abstract

Herpes simplex virus 1 (HSV-1) infection of the cornea leads to a potentially blinding disease, termed herpetic stromal keratitis (HSK) that is characterized by lesions of an immunoinflammatory nature. In spite of the fact that HSK typically presents as a recurrent disease due to reactivation of virus which latently infects the trigeminal ganglia, most murine studies of HSK have employed a primary and not recurrent model of the disease. This report documents the several recurrent models of HSK that have been developed and how data generated from these models differs in some important aspects from data generated following primary infection of the cornea. Chief among these differences is the fact that recurrent HSK takes place in the context of an animal that has a preexisting anti-HSV immune response, while primary HSK occurs in an animal that is developing such a response. We will document both differences and similarities that derive from this fundamental difference in these models with an eye towards possible vaccines and therapies that demonstrate promise in treating HSK.

## 1. Opening Comments

Herpetic stromal keratitis (HSK) is a potentially blinding corneal inflammation that accompanies herpes simplex virus type 1 (HSV-1) infection of the eye. The disease course in HSK begins with a primary infection by HSV followed by a period during which the virus enters latency in sensory and autonomic ganglia. Many studies have shown that clinical disease is the result of a cocktail of inflammatory cells, consisting of PMN's, macrophages, and T cells (both CD4^+^ and CD8^+^) that are recruited to the corneas of patients with HSK [1–4].

 Most animal studies of HSK have focused on primary ocular infection. The major drawback with extrapolating data from primary HSV infection in mice is that it often does not manifest corneal lesions characteristic of human primary or recurrent HSK [5]. We believe that there are four advantages in using a recurrent model of HSK. The first is that recurrent human disease is most often associated with corneal scarring [6, 7]. Second, the clinical profile in the murine recurrent model mimics many of the symptoms observed in human disease [8]. Namely, that primary infection resulted in multiple epithelial dendrites, followed by diffuse stromal opacification, while recurrent infection presented clinical features that included microdendrites, focal stromal opacities, disciform endotheliitis, and corneal neovascularization, which were more similar to those observed in human disease. Third, the model allows reactivation to occur in the context of an immune host. This is also the case in humans where disease takes place in a host that has developed an adaptive immune response against HSV-1. While it is not suggested that such adaptive responses will be identical, they will likely be more similar than extending what occurs following primary infection, where an adaptive response is initially developing (most murine studies), to what is taking place in an “immune host” following reactivation. Finally, a recurrent model lends itself to testing the efficacy of HSV vaccines. Since human disease typically occurs following reactivation, identifying vaccines that are effective when used therapeutically would be very valuable and a recurrent model allows for that evaluation. This is critical as many vaccines which show efficacy in primary HSK models fail when tested in a reactivation paradigm [9].

There are several different protocols that have been used to reactivate mice from latency. One of the first methods used to reactivate mice latently infected was treating mice with the immunosuppressive drug cyclophosphamide (CycloP) [10]. Later Shimeld et al. developed a model in which mice are infected with the McKrae strain of HSV-1 and given passive immunization in the form of pooled serum. The eyes of these latently infected mice are exposed to UV-B irradiation at least 30 days following primary infection to induce reactivation [11–14]. The reason for the addition of passive immunization is that this reduced the high incidence of mortality and also prevented acute HSK, which would lead to permanent corneal damage [13]. It was also noted that the latent infection was restricted to the ophthalmic part of the trigeminal ganglia [13]. When Shimeld et al. [12] and later Laycock et al. [14] compared UV-B irradiation to CycloP + dexamethasone, the incidence of virus shedding from the cornea was greater for the UV-B-treated mice and recurrent disease did was more easily apparent in UV-B-treated mice [12]. Different virus strains were compared for their ability to reactivate, and the McKrae strain of HSV-1 demonstrated the most consistent reactivation phenotype [11–13]. In addition, various strains of mice have been tested for their ability to reactivate following UV-B irradiation and the NIH strain of mice consistently displays the highest rate of reactivation (70–90%) as determined by detection of infectious virus in tear film [9, 14, 15]. In addition, severity of disease also varies between different mouse strains with NIH and BALB/c mice showing severe disease and C57BL/6 mice with much less disease [9, 15, 16]. Other investigators have also developed other means of reactivating a latent HSV-1 infection by using hyperthermia shock [17, 18] and most recently sodium butyrate [19]. The hyperthermia shock model is used to determine reactivation within the infected ganglion neurons and thus is a very good model to study molecular events that occur there following reactivating stress [17, 18]. However it has not been exploited to study corneal disease. The sodium butyrate model, which does result in viral shedding from the corneal surface [19], has also not been exploited to study corneal disease. Thus, while each of these techniques has their own distinct advantages and disadvantages, the UV-B model has been used much more extensively for pathogenic and immunological studies of recurrent HSK. Thus this paper will restrict itself to the reactivation model whereby UV-B light is used to reactivate virus from latency. This was chosen as the vast majority of data concerning the pathogenesis of recurrent disease has used that model.

 As a basis for comparison, a few factors should be mentioned. The first being that different investigators use a variety of strains of HSV-1 when studying primary HSK. The virus strains that have been used for these studies include, but are not restricted to, RE [4, 20, 21], Strain 17 [19], McKrae [8, 15, 22], CH394 [23], and KOS [15, 24]. In addition to a variety of HSV-1 strains being used, various mouse strains have also been used for these studies. Most studies employ C57BL/6 or BALB/c mouse strains or gene-targeted mice that are bred to these two strains. It should be noted that while infections with these various strains of HSV-1 display differences in corneal pathology and neurovirulence, the immunological components of corneal disease are remarkably similar when compared on the basis of virus strain used or the strain of mouse being infected as the following discussion will illustrate.

## 2. Characteristics of Herpetic Stromal Keratitis

As previously mentioned, HSK is at its core an inflammatory disease with chemokines involved in migration of leukocytes to sites of infection and inflammation [25, 26] and cytokines responsible for the activation of cells which mediate the cellular destruction following their activation. Primary models have shown that during disease, a diverse set of host inflammatory cells infiltrate the cornea following HSV-1 infection. Included among these cells are macrophages [8, 9], natural killer cells [27], T cells [8, 20, 28–33], polymorphonuclear neutrophils (PMNs), which are the predominant cell type early during primary infection with HSV-1 [34, 35], and corneal Langerhans Cells [36]. While the exact mechanism for primary HSK has not been conclusively determined, the disease is believed to be the result of the interaction of virus and host immune cells and components and not due to direct viral cytolysis of corneal cells [23]. Since much fewer studies have employed the recurrent model, even less is known about the mechanisms responsible for recurrent HSK.

## 3. Role of Chemokines in HSK

Chemokines are important factors in viral infections [37] and HSK [21, 38–40]. These are small proteins which are made by resident tissue cells and/or immunocompetent cells and whose primary function is to direct the movement or chemotaxis of cells that bear receptors for the chemokine that is being produced. Thus during infection chemokines are released by cells at the site of infection to activate the migration of particular cells bearing appropriate chemokine receptors to that site of infection. Chemokines have also been implicated in activation and polarization of certain immunocompetent cells [41]. Due to these activities, they are potential targets for therapeutic intervention to reduce or prevent disease (see Table 1 for cells responsive to chemokines and references related to HSK). During primary HSK it has been shown that the production of IL-6 stimulates resident corneal cells to produce CCL3 and CXCL2 [42]. Studies have shown that targeting of CCL3 significantly reduces disease [39, 43], suggesting that it plays a role in corneal pathology during primary HSK. In contrast, when CCL3 is neutralized or absent during recurrent HSK, the resulting disease is worse [40]. Likewise, targeting CCL2 reduced primary [44–46] but did not affect recurrent disease [40]. Eo et al. [45] coimmunized mice with a plasmid encoding gB of HSV along with various plasmids containing CCL1, CCL3, CCL4, and CXCL2, which are known to be produced by the cornea [42, 45, 47], to determine if the adaptive immune response generated was affected by the presence of specific chemokines. Their data indicates that the chemokines CCL1 and CCL4 biased immunity to a Th2-like response, while CXCL2 and CCL3 activated a Th1 response. Mikloska et al. observed increased levels of CCL4 and, to a lesser extent, CCL3 and CCL5 in human vesicle fluid from patients with herpetic corneal lesions [48]. Similar studies evaluating CXCL1 expression have shown that infection of corneal-derived cells with HSV-1 leads to significant increases in CXCL1 production, which stimulates neutrophil chemotaxis [47, 49]. It has been reported that CXCL10 is very important in recruiting activated T cells into sites of inflammation [50]. However, CXCL10 has been shown to be a mediator of IL-12-mediated antiangiogenesis during primary HSK [51, 52]. Thus it is possible that CXCL10 might be involved in both promoting disease, by attracting activated CD4^+^ T cells, and in preventing disease by inhibiting corneal angiogenesis. The role of murine CXCL1, CXCL10, and IL-6 play during recurrent HSK has not been determined; however, they are currently being investigated in our laboratory at this time.

## 4. Cytokine Profile Expressed during HSK

 In addition to chemokines, HSK is also the result of the action of various cytokines. Some of these cytokines (IL-1, IL-6, IL-17, and TNF*α*) are found early following virus infection [38, 53–55] and some (IL-1 and TNF*α*) found shortly after reactivation [56]. Staats and Lausch detected increased levels for proinflammatory cytokines, IL-1*α*, and IL-6 following primary HSV-1 infection [53]. In addition, targeting IL-1 during primary HSV-1 significantly reduced disease indicating its central role following acute infection [54]. During recurrent HSK our laboratory also detected significant increases in both IL-1, and TNF*α* [56]. Furthermore, when these cytokines were neutralized in vivo, they were shown to be required for the development of recurrent HSK [56].

 Following this proinflammatory period, a somewhat overlapping set of cytokines (IFN-*γ*, IL-12, IL-17, IL-4 and IL-10) are found later during primary HSK [38, 53, 54, 57, 58], while during this same time period recurrent HSK the cytokines IFN-*γ*, IL-12, IL-4, and IL-10 are reported to be expressed [59–61]. These cytokines can be very informative as they will be indicative of the phenotype of the T cell that is orchestrating the response at that time point. The profile of cytokines made by antigen-specific T cells in an immune response indicates whether it is mediated by Th1, Th2, or Th17 T cells. A Th1 response is characterized by the production of IL-2, IL-12, and IFN-*γ* [62], Th2 cells produce IL-4, IL-5, IL-6, and IL-10 [62], while Th17 cells produce IL-17A, IL-17F, and IL-22 [63]. It has been reported that significant levels of IFN-*γ*, IL-2, and TNF-*α*/*β*, but not IL-4 or IL-10, are found in mouse corneas with primary HSK, suggesting that CD4^+^ T cells in the eye are Th1 [31]. Similar conclusions were derived from studies demonstrating that treatment with anti-IFN-*γ* and anti-IL-2 antibodies, but not with anti-IL-4 antibodies, protected mice from primary HSK [64]. It has been reported that IL-2 not only stimulates Th1 development but also is chemotactic for and maintains the viability of polymorphonuclear neutrophils [65], which are an early component of primary HSK [34, 35]. Further evidence supporting Th1 cell involvement comes from studies using STAT4 KO mice [66]. STAT4 is a transcription factor that is activated by the presence of IL-12 and directs the polarization of T cells to become Th1 cells [67, 68]. When infected with HSV-1, these STAT4 KO mice did not develop significant primary HSK lesions, though they were more susceptible to developing encephalitis [66]. In contrast to these results, it has been shown that when IFN-*γ* KO mice were used to confirm the role of this cytokine during primary HSK surprisingly displayed similar disease to that seen in wild-type mice [67]. This was surprising as one would have expected that such mice would display less corneal disease as IFN-*γ* is an integral cytokine associated with Th1 responses [62, 68]. Interestingly, a very similar phenotype was observed in mice undergoing recurrent HSK [69]. In these studies both IFN-*γ* KO mice and wild-type mice treated with anti-IFN-*γ* were subjected to recurrent HSK and the disease was indistinguishable from recurrent HSK in control antibody-treated wild-type mice [69].

 In a completely different set of studies to determine the role of IL-10 during HSK, it was reported that intraocular treatment of mice with IL-10 reduced corneal disease from 95% to 36% [43, 70]. This treatment, while not effecting the production of IL-1*α* in the cornea, led to a 10-fold reduction in IL-2 and a 50-fold reduction in IL-6 [70]. It has also been reported that IL-10 DNA administration during primary HSK significantly reduced disease [71]. Our laboratory also evaluated the role that IL-10 plays during recurrent HSK, and similar to what was reported during primary HSK [43, 70], lack of IL-10, as determined by neutralizing IL-10 or using IL-10 KO mice, resulted in very severe corneal disease [72]. Furthermore, treating mice with recombinant IL-10 led to significantly reduced corneal disease [72]. Taken together, these studies, both in primary HSK and recurrent HSK, demonstrate that IL-10 is associated with amelioration of disease [72].

 Within the past several years, a new T-cell player has been added to the mix, namely, Th17 cells. These cells were termed Th17 because of their production of the cytokine IL-17 [63]. They have been shown to be involved in autoimmunity and host defense [63]. It was first reported that mice lacking IL-17 receptor displayed reduced neutrophil infiltrate and less corneal disease [73]. This observation was followed by a report that showed that treatment with Resolvin E1 reduced clinical disease and at the same time reduced the numbers of both Th1 and Th17 cells in the cornea [74], further indicating that Th17 cells might be playing a role in primary HSK. That role was further supported when IL-17 KO mice and wild type mice treated with neutralizing antibodies were infected with HSV-1. These studies demonstrated that primary HSK was significantly inhibited thus indicating that both Th1 and Th17 cells likely act in concert to produce the corneal lesions seen during primary HSK [54]. The role that Th17 cells, and thus the cytokine IL-17, play in recurrent HSK has yet to be evaluated.

## 5. T-Cell Functions during HSK

As the aforementioned research implies, T cells are critical to the development of corneal lesions during both primary and recurrent HSK. In fact, T-cell deficient mice do experience HSK [28], unless T cells are adoptively transferred [29]. During primary HSK, the preponderance of data suggests that CD4^+^ T cells of the Th1 subset are the likely mediators of disease [30, 31, 64, 70, 71] while CD8^+^ T cells play a minor role in disease [22, 30, 31, 33, 75] or possibly are involved in reducing disease [15]. Studies attempting to determine if the classical CD4^+^ FoxP3^+^ T_reg_ cells are involved in reducing corneal disease have not conclusively demonstrated that they do so in vivo [4]. Another means of determining the functional role of T cells is the measurement of DTH responses, which are most often mediated by the Th1 subset of CD4^+^ T cells. Using this readout, decreased disease is most often associated with reduced DTH responses [76, 77], though in one case where both Th1 and Th2 responses were allowed to develop, HSK was reduced, but DTH was unaltered [78]. During recurrent HSK, the T-cell profile is somewhat different. Whether by targeting these T-cell subsets by antibody depletion or by using CD4KO and CD8KO mice, these mice expressed a decreased HSK disease phenotype as compared to wild-type and untreated mice [79]. Thus it would appear that both CD4^+^ and CD8^+^ T cells contribute to clinical disease during recurrent HSK [79]. In addition, when the cytokine pattern was investigated, both Th1 and Th2 cytokines were present in the cornea shortly following reactivation [60]. These results should not be surprising as it should be recalled that recurrent HSK occurs in mice that have developed an immune response against HSV-1. That immune response will include antigen-specific CD4^+^ T cells of both Th1 and Th2 subsets as well as HSV-1-specific CD8^+^ T cells. Thus the mixed T-cell infiltrate may, at least in part, explain why focal stromal opacities, rather than the diffuse opacity, as typically seen in primary HSK, characterize recurrent HSK in mice [8]. That said, while there is a report implicating cytotoxic T cells in primary disease [80], recurrent disease is most often associated with strong DTH responses [72, 81].

Since T cells clearly play a central role in mediating disease, identifying those factors that are involved in their activation is very important. A critical factor for activation of T cells is the engagement of costimulatory molecules. The best characterized of these costimulatory interactions are between CD28 or CTLA-4 on the T cell and B7-1 or B7-2 expressed by the APC [82]. Studies have shown that blocking the CD28 interaction with CTLA-4Ig significantly reduced primary HSK by inhibiting Th1 but not Th2 cells [83]. When only B7-2 was blocked, there was a significant reduction in CD8^+^ cytotoxic T-cell response and these mice experienced delayed viral clearance at the cornea [84]. However, these treated mice did not display an impact on corneal disease, suggesting that naïve CD4^+^ T cells are being costimulated via B7-1 [84]. The Hendricks laboratory demonstrated that targeting the CD40-CD154 interaction changed the cellular nature of the inflammatory response but did not result in reduced primary HSK [85], while targeting the OX40-OX40L interaction did not significantly alter primary HSK in any way [86]. When the CD137-CD137L costimulatory pathway was targeted, reduced primary HSK was observed [87]. However, it is interesting to note that this same group later demonstrated that this interaction led to increased CD8^+^ T-cell responses that better controlled HSV-1 infection of the skin [88]. We are currently addressing the role that costimulation plays in recurrent HSK. We hypothesize that since recurrent HSK occurs in an animal that possesses an anti-HSV-1 immune response, we believe that CD28-mediated costimulation will primarily be associated with the initial T-cell activation that occurs shortly after infection and likely is not involved in subsequent stimulation of preexisting anti-HSV-1 T cells that will be present when reactivation of virus from latency occurs. However, it is possible that other costimulatory interactions that occur subsequent to CD28 activation might be important in recurrent HSK. It will be those interactions (CD40-CD154 and CD137-CD137L) that are the focus of ongoing studies in our laboratory.

## 6. Role of T Cells in Maintaining Viral Latency

In addition to their role in mediating corneal disease, T cells have also been implicated in maintaining viral latency in the infected trigeminal ganglia (TG). This role for T cells comes primarily from work done in the laboratory of Robert Hendricks, who reported that CD8^+^ T cells surround latently infected TG neurons in mice infected with HSV-1 [89–92]. In a similar fashion, clinical studies performed on recently deceased individual who know to have infections with HSV-1, found both CD8^+^ and CD4^+^ T cells surrounding neurons that are also stained for HSV-1 antigens [93, 94]. These cells expressed surface markers indicating that they were late effector memory T cells. As was seen in mice, they expressed granzyme markers but no cytolytic function was detected [94]. It has since been reported in murine studies that the production of IFN*γ*, granzyme, and perforin by T cells is important to this maintenance of latency [95, 96]. It was further shown that these infected neurons were not undergoing apoptosis, but that the target of granzyme B released by T cells was the HSV-1 immediate early protein, ICP4 [95], which is essential for further viral gene expression and thus production of infectious virus [97]. More recently the TCR usage of these T cells found in latently infected C57BL/6 TG was characterized [98]. It was demonstrated that the majority were specific for a glycoprotein B peptide, but that CD8^+^ T cells specific for at least 18 other subdominant determinants could be found [98]. One caveat to these studies is that mice lacking CD8^+^ T cells display a similar latency phenotype as normal mice [14]. None the less, taken together, these observations indicate that CD8^+^ T cells are likely involved in maintaining latency but that other factors are also involved.

## 7. Vaccination in HSK

 Many attempts have been made to develop a vaccine that is effective in preventing HSK. While most vaccines prevent primary HSK in animal models when given prior to infection [99–103], they typically fail when delivered therapeutically to prevent recurrent HSK [9, 104]. Nesburn et al. reported that periocular vaccination of latently infected rabbits with recombinant HSV-2 gB/gD in MTP-PE adjuvant resulted in a 2-3-fold decrease in spontaneous corneal viral shedding but had no effect on corneal scarring [105]. They also reported that periocular vaccination with gD1 or gD2 inhibited recurrent dendritic keratitis [106]. Likewise, we and others have shown that vaccination with certain HSV-1 components limits both viral pathogenesis and prevents primary and recurrent corneal disease only when administered prior to infection [9, 104]. If administered following infection these vaccines are ineffective in preventing recurrent disease [9]. These studies illustrate the difficulty of developing effective vaccines for both primary and recurrent infections of HSV. The development of such a vaccine would be of great clinical significance since most patients who present with HSK are latently infected [1, 6, 7]. This was illustrated by a clinical trial in which the vaccine was only effective in women who had never been infected with either HSV-1 or HSV-2 and ineffective in those who had a history of infections with either HSV-1 or HSV-2 [107]. That said, we reported that vaccination with a *vhs*-defective mutant of HSV-1 [108], which had previously been shown to significantly reduce the rate of reactivation [103, 104], would also reduce recurrent HSK when constructed with [109] or without ICP8 [81]. The important thing about these studies was that the vaccine was administered intraperitoneally after infection and thus would have therapeutic value for latently infected individuals. It should be noted that another vaccine construct that consisted of defective *vhs* and ICP8 to which was cloned B7-1 or B7-2 was also effective prophylactically [110] and might also show efficacy when used therapeutically since it is very similar to previous vaccine constructs [81, 109]. Similar results were also seen in studies by Richards et al. who nasally vaccinated latently infected mice with a mixture of HSV-1 glycoproteins and recombinant *E. coli* heat-labile enterotoxin B subunit as an adjuvant [111].

 The mechanism responsible for the therapeutic success of these vaccine constructs has not been fully defined. However, the mechanism could be similar to what was reported in studies involving Theiler's virus- (TMEV-) mediated demyelinating disease in mice. This disease, which is associated with a Th1 antiviral response, was shown to be significantly reduced in infected mice following vaccination with TMEV-coupled spleen cells [112, 113]. These investigators went on to demonstrate that protection is accompanied by a preferential reduction in the Th1 antiviral response and a concomitant increase in the Th2-mediated antiviral response [113]. Thus it appears that protection involves alteration of a Th1-mediated immune response to one primarily mediated by Th2 cells. Results from our vaccination studies using a *vhs* defective replication incompetent virus show similar changes as evidenced by lower DTH responses and increased anti-HSV-1 neutralizing antibody titers in vaccinated mice as compared to mice receiving a control vaccine [81]. Ocular HSV-1 infection typically stimulates an immune response that protects the animal from lethal disease by this virus but also leads to a significant inflammatory response in the cornea that can result in corneal damage. A similar type of immune response also protects latently infected animals that are reactivated from lethal viral disease but can result in significant corneal disease. The mechanism underlying this alteration in the immune response might involve the activation of T regulatory cells. Both conventional CD4^+^ T cells [114, 115] and CD8^+^ T cells [15] have been implicated in regulating the anti-HSV-1 response during primary HSK. However, when Devito and Hendricks tested this, they did not observe an association for T regulatory cells within the cornea and decreased corneal lesions [4]. Thus the case for T regulatory involvement in reducing or resolving primary HSK remains to be established.

## 8. Concluding Remarks

Ocular disease associated with HSV-1 infection (HSK) is the leading cause of infectious blindness and is clearly the result of an immune-mediated inflammatory attack of the cornea. We present data comparing primary and recurrent murine models that are used to study this disease. As should be evident from this discussion, these two model systems, while sharing many characteristics, are not the same. We have learned a lot about this disease from studies of primary HSK. These studies have identified many factors that are responsible for the corneal damage associated with this disease. In fact, these studies have also suggested many potential therapeutic means of treating primary HSK. However, we would argue that without testing these therapies in a recurrent model of HSK, we might be chasing things that ultimately will not prove useful in the clinic. Consequently, due to the paucity of studies investigating recurrent HSK, much research remains to be performed. Particularly concerning those aspects of primary HSK that are reflected in recurrent HSK and also those things that are not, we contend that a better understanding of the immunological factors, both cellular and cell-free, that underlie the development of recurrent HSK will enable us to identify potential therapies that might prove to be most effective in treating human HSK.

## Figures and Tables

**Table 1:** 

Chemokine	Chemotactic activity	Reference to HSK
CCL1 (Kit ligand)	Monocytes, NK cells, DC	[45]
CCL2 (MCP-1)	Monocytes, T cells, DC	[40, 44–46]
CCL3 (MIP-1*α*)	Neutrophils	[39, 40, 42, 43, 45, 48]
CCL4 (MIP-1*β*)	Monocytes, NK cells	[45]
CCL5 (Rantes)	Eosinophils, NK cells, T cells	[48]
CXCL1 (KC)	Neutrophils	[47, 49]
CXCL2 (MIP-2)	Neutrophils	[42, 45]
CXCL10 (IP-10)	Monocytes, NK cells, T cells, DC	[50–52]
